# Supplementary material for: Patterns of Intron Gain and Loss in Fungi
Source: PLoS Biol. 2004 Nov 30;2(12):e422. doi: 10.1371/journal.pbio.0020422 (PMC532390; doi:10.1371/journal.pbio.0020422)
Supplement: Table S1 — Also available at http://genes.mit.edu/NielsenEtAl/. (4.3 MB ZIP). [file pbio.0020422.st001.zip › NielsenEtAl/html/386.html]

AN7388.1.NCU05770.1.MG04337.1.FG02974.1


```
 CLUSTAL W (1.82) Multiple Sequence Alignments - Introns Inserted


Sequence 1: NCU05770.1	753 aa
Sequence 2: MG04337.1	738 aa
Sequence 3: AN7388.1	739 aa
Sequence 4: FG02974.1	738 aa
Alignment Length: 764 aa
Number Identitical Residues: 380 aa
Alignment Score (without introns) 19844


MG04337.1 	MG--ECPLRT--ANVAGGGTRNRDWWPNTLKLNILRQHTEATNPYDPNFDYAEAFKSLDY
NCU05770.1	MS--ECPVRK--SNVGGGGTRNHDWWPAQLRLNILRQHTPVSNPLDKDFDYAAAFKSLDY
FG02974.1 	MSESKCPVAHKIPNVAGHGVTHQKWWPESLKTNILRQHSNVTNPYGENFSYAEAFKSLDY
AN7388.1  	MGSNECPYSRQNANIGGGGQNNRDWWPDDLKLNILRQHNSVSNPLDKGFDYTAAFNSLDY
          	*...:**   . .*:.* *  ::.***  *: ******. .:** . .*.*: **:****

MG04337.1 	EGLKKDLRALMTDSQEYWPADFGHYGGLFVRMAWHSAGTYRVMDGRGGGGQ~GQQRFAPL
NCU05770.1	EGLKKDLTKLMTDSQDWWPADFGHYGGLFIRMAWHSAGTYRVTDGRGGGGE~GQQRFAPL
FG02974.1 	NALKEDLRKLMTDSQDWWPADFGHYG----------------------GLM0GQQRFAPL
AN7388.1  	FGLKRDLEALMTDSQDWWPADFGHYGGLFIRMAWHSAGTYRVFDGRGGGGQ~GQQRFAPL
          	 .**.**  ******::*********.     :  ::.:    .. ..*   ********

MG04337.1 	NSWPDNVSLDKARRLLWPIKQKYGNKISWADLMLLTGNVALEDMGFKTFGFAGGRPDTWE
NCU05770.1	NSWPDNVSLDKARRLLWPIKQKYGNKISWSDLLLLTGNVALESMGFKTFGFAGGRPDTWE
FG02974.1 	NSWPDNVSLDKARRLLWPVKQKYGNKISWADLIVLAGNVALEDMGFKTLGFAGGREDTWE
AN7388.1  	NSWPDNVSLDKARRLLWPIKQKYGSKISWADLLILAGNVALESMGFKTFGFAGGRSDTWE
          	******************:*****.****:**::*:******.*****:****** ****

MG04337.1 	ADESTYWGGETTWLGNEVRYSSGNEGHKESGVIDGSESKKGHKDIHTRDLEKPVSAAHMG
NCU05770.1	ADESVYWGAETTWLGNEDRYSEGQEGHEGHGVVQGDESKKQHTDIHNRDLQSPLASSHMG
FG02974.1 	ADEATYYGGEDTWLGNDVRYSNGNKGTTKPGATDSDQ--TEHKDIHNRDLEKPLAAAHHG
AN7388.1  	ADQSVFWGGEKEWLGNDVRYLNG-------------------------ELDNPLAASHMG
          	**::.::*.*  ****: ** .*                         :*:.*::::* *

MG04337.1 	LIYVNPE~GPDGIPDPVAAARDIRTTFSRMAMNDEETVALIAGGHTVGKTHGAAPSDNVG
NCU05770.1	LIYVNPE~GPDGIPDPVASAKDIRVTFGRMAMNDEETVALIAGGHSFGKTHGAGPTHHVG
FG02974.1 	LIYVNPE1GPDGNPDPVAAAHDIRETFGRMAMNDEETVALIAGGHTVGKTHGAGSTDHVG
AN7388.1  	LIYVNPE~GPNKNPDPVLAAKDIRITFGRMAMNDEETVALIAGGHTFGKTHGAGPATHLG
          	******* **:  **** :*:*** **.*****************:.******..: ::*

MG04337.1 	PEPEAAPIENQGLGWSNKHGSGKGPDTITSGLEVIWTKEPAKFTMNYLEYLFKYEWELTK
NCU05770.1	KEPEAAPIEHQGLGWANSFGQGKGPDTITSGLEVTWTPTPTKWGMGYLEYLYKFDWEPTK
FG02974.1 	PEPEAADLAQQGLGWSNSYKSGKGPDTTTSGIEVTWTSTPVKWSHDYLKYLFQFEWELTK
AN7388.1  	KEPHGAGIELQGLGWESGFESGTGRHAITSGLEVIWTKTPTKWSNQFFEYLFKYDWELTK
          	 **..* :  ***** . . .*.* .: ***:** **  *.*:   :::**::::** **

MG04337.1 	SPAGANQWVAKNAEEFIPDAFDPSKKHKPRMLTTDLSLRFDPEYEKISRRFLENPEQFKD
NCU05770.1	SPAGANQWVAKNAEPTIPDAYDPNKKKLPTMLTTDIALRMDPAYDKICRDYLANPDKFAD
FG02974.1 	SPAGAHQWVAKDAEATVPHAYDSSKKQRPSMLTTDLSLRFDPAYEKISRRFLENPQEFND
AN7388.1  	SPAGAHQYVAKGVEPFIPDPFDPSIKHPPRMLTTDLSLRYDPEYEKISRRFLENPDQFAD
          	*****:*:***..*  :*..:*.. *: * *****::** ** *:**.* :* **::* *

MG04337.1 	AFARAWFKLLHRDMGPRSRWLGPEVPKETLLWEDPIPTPDHPIIDGSDVDSLKKAILATG
NCU05770.1	AFARAWFKLLHRDMGPRTRWIGPEVPSEILPWEDYIPPVDYQIIDDNDIAALKKEILATG
FG02974.1 	AFAKAWFKLTHRDMGPRDRYLGPEVPKEIFSWQDPVPERDYKVVDDGDISAIKNEILKSG
AN7388.1  	AFARAWFKLTHRDVGPRVLYQGPEVPSEVLIWQDPVPPLDHPVIDNDDIATLKKAILNSG
          	***:***** ***:***  : *****.* : *:* :*  *: ::*..*: ::*: ** :*

MG04337.1 	VAPSKLIQTAWASASTFRGGDKRGGANGARIRLEPQNKWEVNNPQQLAEVLKALEGVKAD
NCU05770.1	VAPKKLIFVAWSSASSFRGSDKRGGANGARIRLAPQNEWKVNDPSTLREVLAALESVQQK
FG02974.1 	IDVSKLVSTAWASASTFRGSDLRGGANGARLRLEPQKNWEVNNPSQLGQVLNGLEKIQKN
AN7388.1  	ISHTDLFSTAWASASTFRGSDKRGGANGARIRLSPQKNWKVNSQPWLSESLAALEKIQKQ
          	:  ..*. .**:***:***.* ********:** **::*:**.   * : * .** :: .

MG04337.1 	FE--KSGKK-VSIADLIVLAGVAAVEQAA-----GVPVPFTPGRGDATQEQTDVESFTHL
NCU05770.1	FNDSSSGKK-VSLADLIVLGGVAALEQAS-----GLVVPFTPGRNDATQEHTDVHSFTHL
FG02974.1 	FNDSQSNGKAISLADVIVLAGSAAVEKAAKDAGVNVTVPFTPGRTDATQDQTDVQSVAHL
AN7388.1  	FNDAQSTDKRVSLADLIVLAGAASLEKAARDAGHNVSVSFTPGRTDATQEQTDVDSFNNL
          	*:.:.*  * :*:**:***.* *::*:*: .:. .: *.***** ****::***.*. :*

MG04337.1 	EPAADAFRNYGKGTSRVTTEQIMVDRAQQLTLTAPELTVLVGGLRVLGANYDGSSHGVWT
NCU05770.1	EPHADGFRSYGKGTKRVRTEQFLIDRASLLTLSAPELTALIGGLRVLEANYDGSSYGVLT
FG02974.1 	EPAADGFRNYGASTDRVKLEHMLIDRAQLLTLSVPEITALVGGLRALNANWDGSSHGILT
AN7388.1  	EPIADGFRNYGRGTPRVLTEDFLIDKAQLLNLSPPELTVLIGGLRVLNNNYDRSNLGVFT
          	** **.**.** .* **  *.:::*:*. *.*: **:*.*:****.*  *:* *. *: *

MG04337.1 	DKPGKLTNDFFVTLLDPYTSWKSVD--GEVFEGTNSKS-GKKLTGTRADLVFGSHSELRA
NCU05770.1	KTPGKLTNDYFVNLLDTNTAWKAADNEGEVFIGYDRKTHDKKWTATRADLIFGAHAELRA
FG02974.1 	KRPGVLSNDFFVNLLDISTEWKPVGN-GDLFEGIDRKSGNKKWTGTRVDLVFGSHAELRA
AN7388.1  	KRPGQLTNDFFVNLLDMGVQWKPADDTNEIFIGSDRKTGQARWKASRADLVFGSHAELRA
          	. ** *:**:**.***  . **.... .::* * : *:   : ..:*.**:**:*:****

MG04337.1 	LAEVYGSADGQQKFTKDFVAAWDKVMNLDRFDVRR-----------
NCU05770.1	LAEVYAAVDGEEKFKRDFVAAWHKVMNLDRFDLKQEGRGQNAPKL-
FG02974.1 	TAETYAEAGGSEKLVRDFVAAWTKVMNLDRFDVKN-GKAPNVSPRL
AN7388.1  	ISEVYGSSDGEAKFVKDFVAAWEKVSNLDRFDLKQTGLAQRIKPQL
          	 :*.*.  .*. *: :****** ** ******::. . .
```
